# Women’s attitudes towards physical intimate partner violence in India: Trends, patterns, and determinants

**DOI:** 10.1371/journal.pone.0318350

**Published:** 2025-03-12

**Authors:** Shreemoyee Shreemoyee, Punarjit Roychowdhury, Gaurav Dhamija

**Affiliations:** 1 Department of Liberal Arts, Indian Institute of Technology Hyderabad, Sangareddy, Telangana, India; 2 Shiv Nadar University, Greater Noida, Uttar Pradesh, India; 3 Global Labor Organization, Essen, Germany; 4 Centre for Development Economics and Sustainability, Monash University, Clayton, Australia; 5 Center for Research on Economics of Climate, Food, Environment and Energy, Indian Statistical Institute, New Delhi, India; University of Gour Banga, INDIA

## Abstract

Women’s attitudes towards physical intimate partner violence are a major determinant of the likelihood of their exposure to physical intimate partner violence. In this study, we scrutinize the third, fourth, and fifth rounds of the National Family Health Survey using descriptive analyses and logistic regression models to understand the trends, patterns, and drivers of women’s attitudes towards physical intimate partner violence across various demographic and socioeconomic groups in India. Our findings reveal a noticeable decline in the level of women’s acceptability of physical intimate partner violence over the past 15 years, albeit at a slow pace. Furthermore, the study demonstrates that the acceptability of physical intimate partner violence is more prevalent among women from demographically and socioeconomically disadvantaged backgrounds. This includes women who marry at a young age, have no formal education, are exposed to interparental violence, belong to lower caste or tribal communities, exhibit poor wealth status, and reside in rural areas. The findings suggest a need for targeted policy interventions focusing on enhancing educational opportunities and promoting socioeconomic equity, particularly within demographically and socioeconomically disadvantaged groups.

## Introduction

Gender-based violence, particularly instances of physical and sexual intimate partner violence (IPV), represents a significant global concern. The World Health Organization (WHO) defines IPV as “any behavior within an intimate relationship that causes physical, psychological, or sexual harm to those in the relationship,” including acts of physical aggression, sexual coercion, psychological abuse, and controlling behaviors [1]. Statistics indicate that one in three women has encountered physical or sexual violence from their partners at some point in their lives [1]. Physical intimate partner violence (PIPV) is defined as the intentional use of physical force by the intimate partner with the potential for causing death, disability, injury, or harm [2]. It includes, but is not limited to, the following acts of physical harm: scratching, pushing, shoving, throwing, grabbing, biting, choking, shaking, hair-pulling, slapping, punching, hitting, burning, use of a weapon (gun, knife, or other object); or a combination of these acts [3]. PIPV stands out as the most prevalent form of spousal abuse, exerting severe consequences on various aspects of women’s well-being [4,5] as well as their children [6,7].

An alarming facet of IPV is the prevalence of regressive attitudes toward it in certain sections of society [8–10]. In these contexts, women may justify IPV in situations where they fail to meet their responsibilities or deviate from traditional gender norms [11]. The National Family Health Survey-5 (NFHS-5) report [12] reveals that 45% of women in India justify PIPV. Recent evidence from India indicates that women’s beliefs about PIPV play a significant role in determining the incidence of IPV. Women who believe that PIPV perpetration is justified face a 37.4 percentage point higher likelihood of experiencing IPV compared to those who do not hold such beliefs [8]. As the acceptability of PIPV increases among women, the likelihood of their exposure to IPV also rises by 37.1 and 38.2 percentage points in urban and rural areas, respectively [9]. These results likely suggest that reducing women’s acceptability of PIPV is crucial for mitigating their exposure to IPV. Therefore, from a policy perspective, it is imperative to identify sections of Indian women exhibiting higher levels of acceptability of PIPV and monitor changes in these levels within these groups over time.

A limited number of studies have specifically examined the determinants of women’s acceptability of PIPV in India. However, these studies either exhibit a certain degree of obsolescence [3,10,13–15] or have relied on data sourced exclusively from particular states or regions within India [16–18]. In light of this, our study takes a novel approach by leveraging data from women aged 15–49 years in the three most recent rounds of the National Family Health Survey (NFHS-3, NFHS-4, and NFHS-5) conducted in 2005–06, 2015–16, and 2019–21. We aim to address the following questions with respect and thoroughness: (1) What is the level of acceptability of PIPV among women across various subgroups characterized by demographic, socioeconomic, and regional factors? (2) Has the level of acceptability of PIPV among women in these subgroups changed over the past 15 years? (3) What are the most significant determinants influencing women’s acceptability of PIPV?

We employ descriptive analyses to calculate the patterns and trends of women’s acceptability of PIPV. This analysis encompasses the overall sample of women aged 15–49 years, as well as various subgroups distinguished by factors such as age, education, marital status, age at first cohabitation, employment status, access to a bank account, media exposure, experience of interparental violence, husband’s education, religion, social group, household wealth, place of residence, and states of India. Multivariable logistic regression analysis is applied to delve deeper into the dynamics. This method allows us to get a comprehensive understanding of the specific roles these correlates play in either increasing or decreasing the likelihood of women’s acceptability of PIPV.

We find evidence of a decreasing trend in the level of women’s acceptability of PIPV over the past 15 years. However, this decline seems to have occurred at a relatively sluggish rate. Moreover, higher levels of acceptability are observed among women hailing from demographically and socioeconomically disadvantaged backgrounds. These include women who marry at a very young age, lack formal education, have exposure to interparental violence, belong to scheduled castes, backward classes, or tribal communities, possess limited wealth, and reside in rural areas. These results underscore the importance of targeted policy interventions that prioritize improving educational opportunities and promotion of socioeconomic equity, particularly within demographically and socioeconomically disadvantaged groups. Such measures are crucial to reduce the acceptability of PIPV in society further and address the persistent challenges observed in specific vulnerable groups.

## Literature review

### Theoretical framework

Patriarchal societies are characterized by deeply entrenched gender norms and expectations that dictate women’s roles and responsibilities [19]. These norms emphasize duties such as properly preparing food and childcare, seeking permission before leaving the house, obeying the husband and in-laws, and fulfilling marital sexual obligations. Transgressing from these pre-established gender roles often perpetuates PIPV in such patriarchal societies [19–23]. Given the pervasive influence of these patriarchal norms, understanding the factors that shape women’s attitudes towards PIPV becomes critical.

Various theories have been proposed to understand women’s attitudes towards PIPV. Rani et al. (2004), in their pioneering work [19], use the social learning theory [24,25] and the ecological framework [26] to understand the determinants of attitudes towards PIPV. The ecological framework is a theoretical model proposed by Heise (1998) to explain how IPV is a result of a complex interplay of factors at different levels. These levels encompass personal history at the individual level, interpersonal relationships within families, social and institutional structures at the community level, and broader cultural norms at the societal level. [26]. The social learning theory posits that behaviors, including adherence to patriarchal gender roles, are learned through observation or imitation of role models within familial and social groups [19,27]. The division of labor between men as breadwinners and women as homemakers or child bearers is reinforced intergenerationally through this social learning process. This theory suggests that the myth of male superiority, rooted in patriarchal ideology, lies at the core of the acceptability of PIPV [19]. However, these attitudes are not fixed and can evolve over an individual’s lifespan due to individual, family, community, or societal factors outlined by the ecological framework. These factors influence the acceptability of PIPV by a) creating conflicts between the reality of lived experiences, such as women’s employment, and the notion of male superiority, b) exposure to impartial social networks, such as those fostering educational attainment, and c) access to non-conformist ideas disseminated via modern media and education [10,19,27].

On the other hand, the normalization theory describes how women rationalize the male perpetration of violence as a consequence of their failure [28,29]. In India, evidence suggests that IPV is considered a normal part of marital or intimate relationships [18]. Women from such communities often adhere to the ‘just world hypothesis,’ where individuals believe outcomes are a direct result of their actions. Therefore, women may internalize blame, perceiving violence as a deserved punishment for their inability to conform to prescribed roles [30].

The resource theory provides an economic lens to understand the acceptability of PIPV among women. According to this theory, women with lower economic status — manifested through limited income, education, occupational opportunities, or household wealth — are more likely to depend financially on their abusive partners [31,32]. This dependency increases their vulnerability to justifying PIPV [33,34], as leaving or challenging an abusive relationship often entails significant economic costs [35].

### Determinants of women’s attitudes towards IPV

Existing literature on the correlates of women’s attitudes towards IPV in developing countries predominantly focuses on individual, household, and community factors. Studies have consistently found that women’s age, age at marriage, education, and husband’s education levels are negatively associated with the likelihood of justifying IPV [10,14,28,29,36,37]. These relationships may be attributed to an accumulation of status within families, enhanced awareness and self-confidence, and greater exposure to progressive views [38–40]. Similarly, greater access to mass media is linked to a decline in the acceptability of PIPV [13,41]. Media exposure is instrumental in raising awareness about women’s rights and showcasing lifestyles beyond individuals’ immediate surroundings. This exposure can significantly influence women’s perceptions of fair treatment within relationships and promote a deeper understanding of egalitarian ideals, thereby challenging traditional norms that justify PIPV.

The relationship between women’s engagement in formal employment and their attitudes toward PIPV is complex. Some studies suggest that employment reduces the justification of IPV by enhancing women’s bargaining power and fostering economic independence [42]. However, other research studies highlight that employed women may exhibit higher levels of acceptability toward PIPV. This counterintuitive finding is often explained by feelings of guilt associated with not fulfilling traditional domestic roles, which may lead women to internalize blame for incidents of PIPV [10,22].

Another critical determinant is the intergenerational transmission of attitudes toward violence. Women who witness interparental violence as children—such as their mothers being beaten by their fathers—are more likely to normalize IPV in their relationships. Such experiences shape perceptions of IPV as an inherent and acceptable aspect of marital dynamics, thereby perpetuating cycles of victimization [9,37].

Household wealth is another factor correlated with the justification of PIPV [10,43]. In lower-income households, PIPV might be normalized as a coping mechanism for stress or perceived as an unavoidable aspect of life [20,44]. Moreover, women from urban areas tend to demonstrate lower tolerance for PIPV than their rural counterparts. This pattern can be attributed to the greater exposure to egalitarian and modern values prevalent in urban settings [10,45,46].

Our study aligns with at least two key strands of literature. The first strand explores the determinants of women’s attitudes towards IPV, with a particular focus on identifying critical factors influencing these attitudes. The foundational work in this area has been discussed above. Second, we add to the literature that examines the determinants of women’s exposure to IPV across various countries. A substantial body of empirical literature examines the association between women’s exposure to IPV and various individual, spousal, and household characteristics [26,47,48]. Moreover, studies in this domain have analyzed a broad range of environmental determinants, including macroeconomic and labor market conditions [22,49–55], cultural and social norms [56–58], human capital [59,60], gender ratios [61], age at marriage [62], violation of educational hypergamy [63], decision to use contraceptives [64], and divorce laws [65,66].

This strand of literature also delves into behavioral motives for IPV [67,68] and instrumental violence, where perpetrators use abuse to manipulate victims’ behavior or extract resources from their families [69–71]. Additionally, several studies have investigated the impact of government policies on the prevalence of IPV, including law enforcement measures [72,73] and welfare or cash-transfer programs [74–77].

### Data

This paper uses data from the third, fourth, and fifth rounds of the National Family Health Survey (NFHS-3, NFHS-4, and NFHS-5) conducted in 2005–06, 2015–16, and 2019–21. The NFHS is a nationally representative cross-sectional dataset for India and part of the global Demographic Health Survey (DHS) program. It is conducted by the International Institute for Population Sciences (IIPS) under the stewardship of the Ministry of Health and Family Welfare (MoHFW), Government of India. The NFHS-3 collected information from 28 states and the National Capital Territory of Delhi, covering a nationally representative sample of 1,24,385 women aged 15–49 and 74,369 men aged 15–54 [78]. To provide district-level estimates, the NFHS-4 covered a large sample size of 6,99,686 women and 1,12,122 men from 29 states, 7 union territories, and 640 districts of India [79]. The NFHS-5 covered 7,24,115 women and 1,01,839 men from 28 states, eight union territories, and 707 districts of India [12].

#### Study participants.

**Fig 1:**
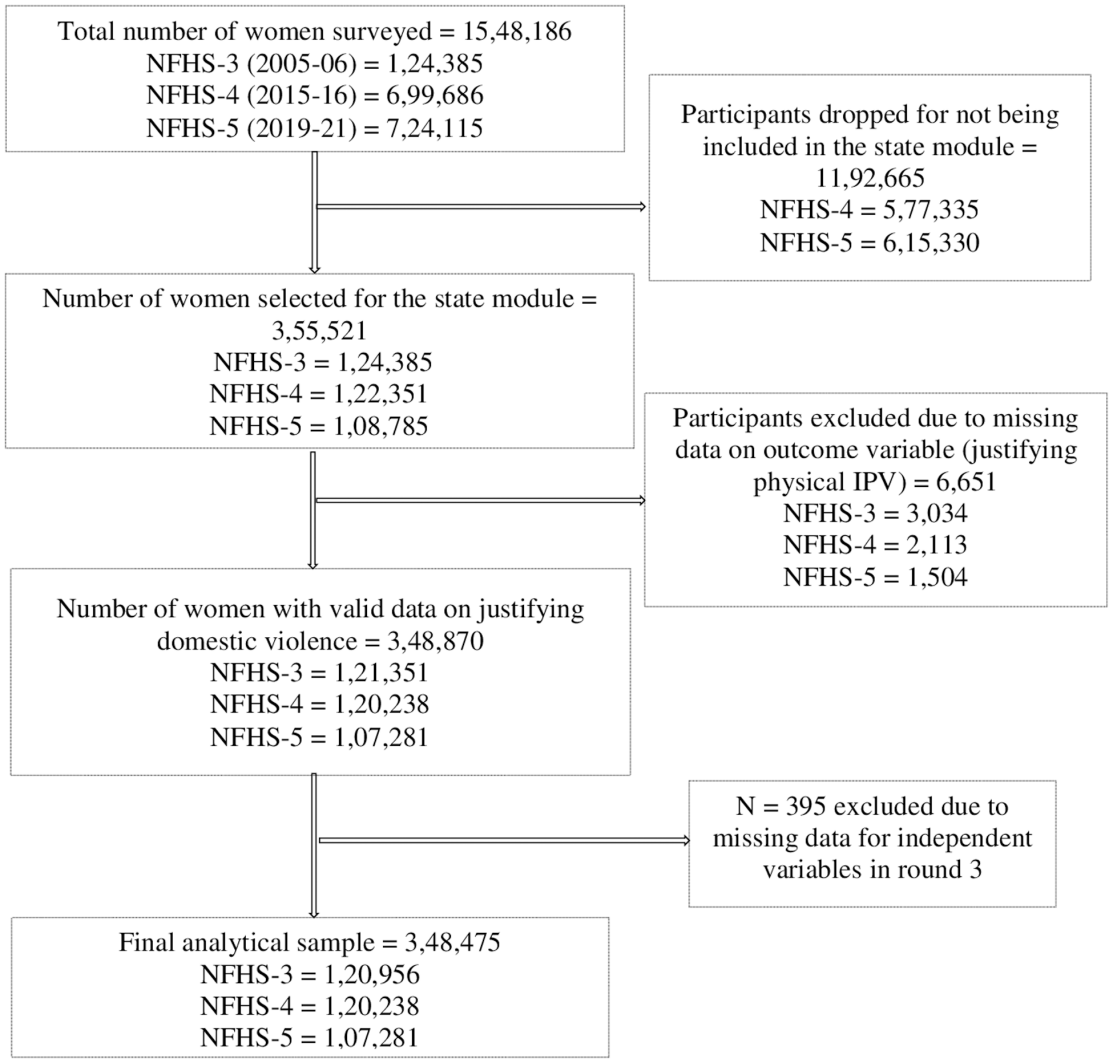
Schematic representation of the women included in the final analytical sample.

We use the women’s data from the NFHS-3, NFHS-4, and NFHS-5 datasets with information on 1,24,385, 6,99,686, and 7,24,115 women, respectively. From this surveyed sample, our analytical sample consists of 1,20,956, 1,20,238, and 1,07,281 women from rounds 3, 4, and 5 who are selected for the state module and have complete data on the outcome variable and explanatory variables. Fig 1 gives a detailed schematic representation of the women included in the analytical sample.

### Methodology

#### Outcome variable.

Our outcome variable is women’s acceptability of PIPV. NFHS captures information on the attitudes towards PIPV through a set of questions on whether the respondent justifies husbands (or intimate partners) beating their wives if she: (a) goes out without telling the husband, (b) neglects her children, (c) argues with the husband, (d) refuses to have sex with the husband, (e) burns the food, (f) is unfaithful, (g) is disrespectful to her in-laws. Our outcome variable, acceptability of PIPV, takes a value of one if a woman justifies wife-beating for at least one of the above reasons and zero otherwise.

As a robustness check, we use an alternative measure of acceptability of PIPV. Our alternative outcome variable takes the value 1 if the respondent justifies wife-beating for at least 4 reasons and 0 otherwise. Results remain qualitatively unchanged except for women’s exposure to media, which shows a negative association (AOR 0.88; 95% CI, 0.85–0.91) with women’s acceptability of PIPV. Results are available on request.

#### Explanatory variables.

Our analysis uses a range of individual and household-level demographic and socioeconomic characteristics as explanatory variables. At the individual level, we include women’s age (15–19, 20–29, 30–39, and 40–49), education (no education, primary, secondary, and higher), marital status (never married, currently married, and single—single women include married women whose gauna has not been performed, widowed, divorced, separated, and deserted women), age at first cohabitation (less than 18, 18–21, more than 25, and others—others include women who are never married, married but gauna has not been performed, widowed, divorced, separated, and deserted), employment status (not working, employed in an agricultural occupation, employed in other occupations—other occupations include professional, technical, managerial, clerical, sales, household, and domestic services, skilled and unskilled manual labor, and other services, and don’t know/missing), access to bank account (no and yes), media exposure (no and yes), experience of inter-parental violence (no, yes, and don’t know/missing), and spousal education (no education, primary, secondary, higher, never married, and don’t know/missing).

At the household level, we include religion (Hindu, Muslim, Christian, and Others including Sikh, Buddhist, Jain, Jewish, Parsi, no religion, or any other religion), social group (Upper Caste [UC], Scheduled Castes [SC], Scheduled Tribes [ST], Other Backward Classes [OBC], and don’t know/missing), wealth (poorest, poor, middle, rich, and richest), and place of residence (urban and rural) as the markers of demographic and socioeconomic status. We also conduct a spatiotemporal analysis of women’s acceptability of PIPV across India’s geographically and culturally diverse regions (North, West, Central, East, North-East, and South).

### Statistical analysis

**Fig 2:**
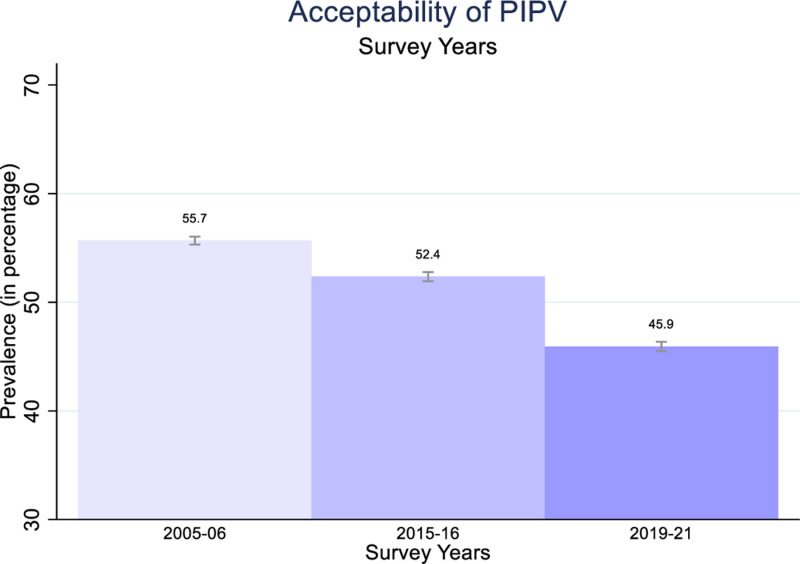
Women’s acceptability of PIPV in 2005–06, 2015–16, and 2019–21, India.

**Fig 3:**
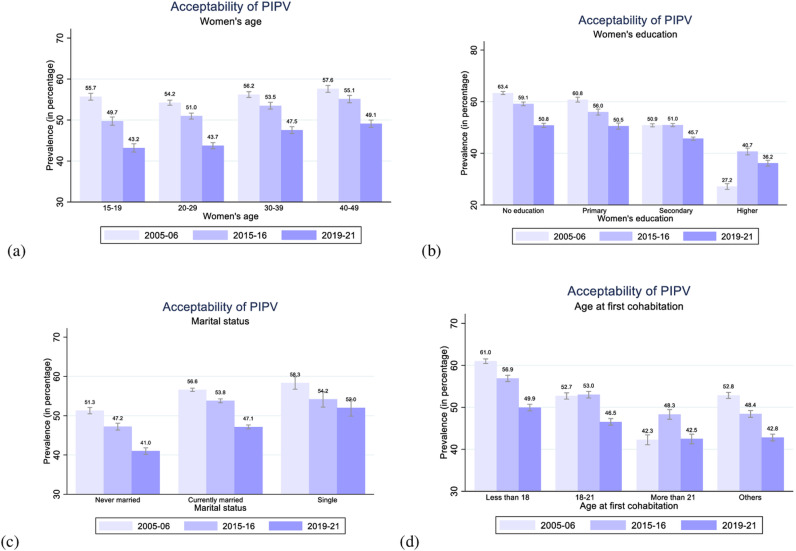
Women’s acceptability of PIPV by their age, education, marital status, and age at first cohabitation in 2005–06, 2015–16, and 2019–21, India.

**Fig 4:**
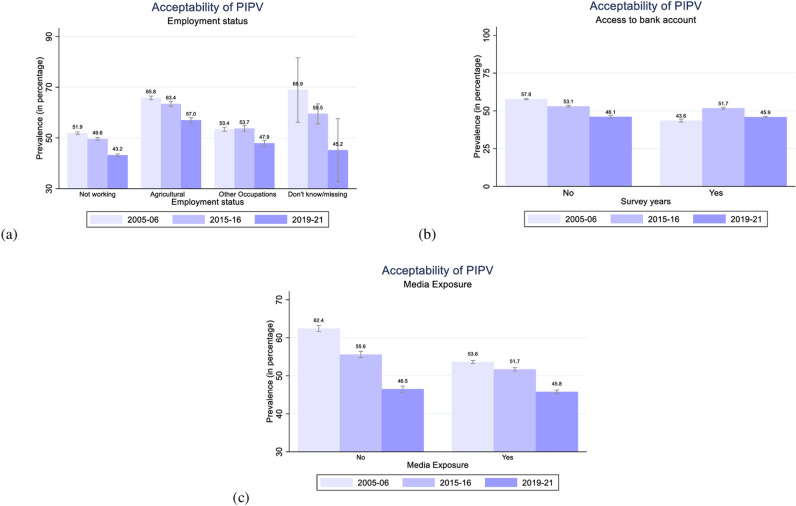
Women’s acceptability of PIPV by their employment status, access to bank account, and media exposure in 2005–06, 2015–16, and 2019–21, India.

**Fig 5:**
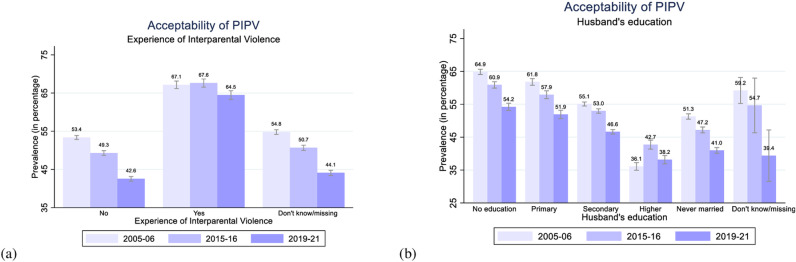
Women’s acceptability of PIPV by their experience of interparental violence and husband’s education in 2005–06, 2015–16, and 2019–21, India.

**Fig 6:**
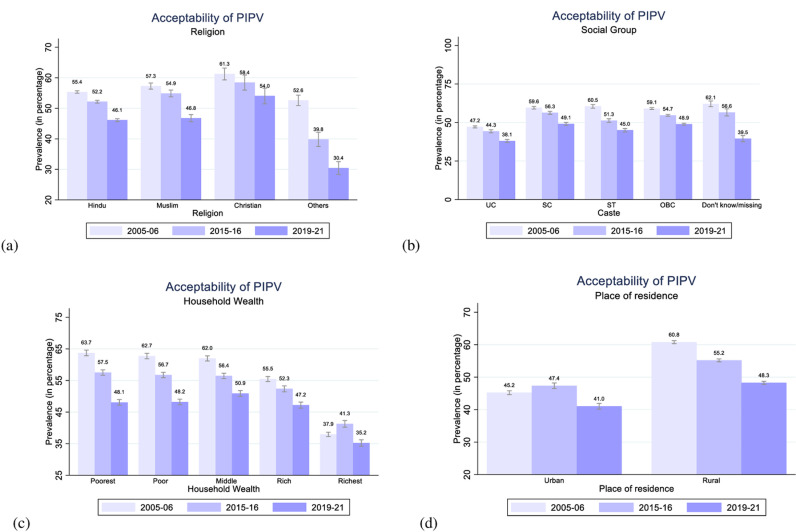
Women’s acceptability of PIPV by religion, social group, household wealth, and place of residence in 2005–06, 2015–16, and 2019–21, India.

**Fig 7:**
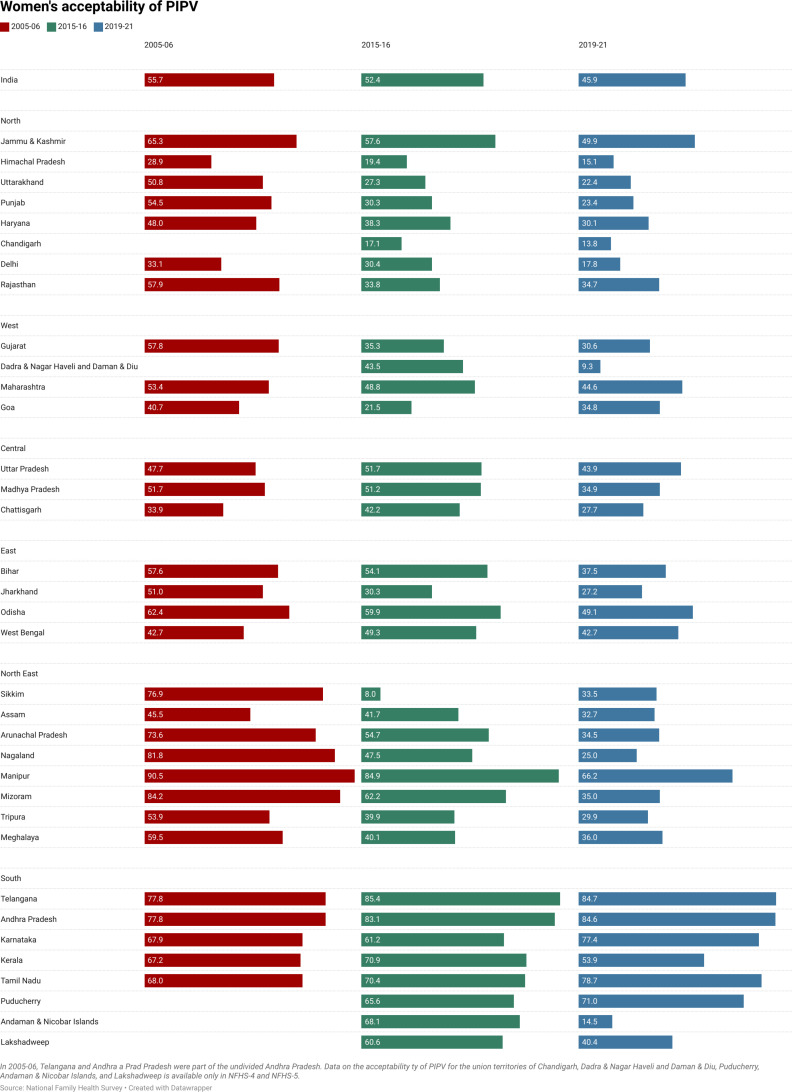
Women’s acceptability of PIPV by different states and union territories in 2005–06, 2015–16, and 2019–21, India.

Table 1 provides the distribution of demographic and socioeconomic characteristics within the pooled sample as well as for each survey year, accounting for the sample weights. To quantify the trends and patterns of the level of women’s acceptability of PIPV, weighted estimates are calculated for the overall sample (Fig 2) and by different demographic and socioeconomic characteristics (Figs 3–6) in all three rounds of the NFHS. The trends and patterns of women’s acceptability of PIPV in different states of India are presented in Fig 7. Based on the referee’s suggestion, we have also presented the trends and patterns of women’s exposure to physical, emotional, and sexual violence by different demographic and socioeconomic characteristics for all three rounds of the NFHS in S1 Appendix Figs A1–A18. Next, logistic regression analysis is used to identify the significant determinants of women’s acceptability of PIPV, accounting for survey design characteristics and sample weights. Adjusted odds ratios (AOR) and a 95% confidence interval, adjusted for the survey round fixed effects and region fixed effects, are estimated for the pooled sample of three survey rounds (Table 2). We have also presented the adjusted odds ratios and a 95% confidence interval based on the analytical sample from each survey round of NFHS in Table 2.

**Table 1:** Percentage distribution of demographic and socioeconomic characteristics of women aged 15–49, India.

Characteristic	NFHS-5	NFHS-4	NFHS-3	Pooled
	(1)	(2)	(3)	(4)
All India	100	100	100	100
**Women’s Age**				
15–19	16.5	16.9	19.4	17.7
20–29	32.7	33.9	34.9	33.9
30–39	27.8	27.1	27.2	27.4
40–49	23	22.1	18.5	21.1
**Women’s Education**				
No education	22.6	26.3	40.8	30.2
Primary	11.8	12.3	14.7	13
Secondary	49.9	48.0	37.2	44.8
Higher	15.7	13.4	7.3	12
**Marital Status**				
Never married	23	22.0	19.1	21.3
Currently married	72.4	73.3	75.6	73.8
Others	4.6	4.7	5.4	4.9
**Age at First Cohabitation**				
Less than 18	29.9	31.4	45.1	35.7
18–21	28	28.1	22.8	26.2
More than 21	14.5	13.8	7.7	11.9
Others	27.6	26.7	24.4	26.2
**Employment Status**				
Not working	69.5	69.6	57.2	65.2
Agricultural	14.1	14.7	25.2	18.2
Other Occupations	16.4	14.5	17.6	16.2
Don’t know/missing	0.1	1.2	0	0.5
**Access to Bank Account**				
No	21.3	46.9	84.8	52.2
Yes	78.8	53.1	15.2	47.8
**Media Exposure**				
No	22.7	17.5	23.3	21.1
Yes	77.3	82.5	76.7	78.9
**Experience of Interparental Violence**				
No	51.7	49.5	50.3	50.5
Yes	12.9	14.0	12.9	13.3
Don’t know/missing	35.3	36.5	36.8	36.3
**Husband’s Education**				
No education	13.7	15.1	21.9	17
Primary	11.2	11.6	12.9	12
Secondary	40	40.1	36.2	38.7
Higher	11.9	11.0	9.1	10.6
Never married	23	22.0	19.1	21.3
Don’t know/missing	0.3	0.2	0.8	0.5
**Religion**				
Hindu	80.8	80.4	80.7	80.6
Muslim	14	14.3	13.6	14
Christian	2.3	2.5	2.5	2.4
Others	2.9	2.9	3.3	3.0
**Social Group**				
UC	19.9	23.1	31	24.9
SC	21.7	19.5	18.7	19.9
ST	9.1	9.0	8.1	8.7
OBC	43.8	44.1	39.2	42.3
Don’t know/missing	5.5	4.3	3	4.2
**Household Wealth**				
Poorest	18.4	16.0	17.5	17.3
Poor	20.4	18.9	19	19.4
Middle	20.6	20.8	20.1	20.5
Rich	20.7	21.8	21	21.2
Richest	19.9	22.5	22.4	21.7
**Place of Residence**				
Urban	32.3	35.9	32.8	33.7
Rural	67.7	64.1	67.2	66.3
Observations	107281	120238	120956	348475

Notes: Columns (1), (2), and (3) present the percentage distribution of women’s socioeconomic and demographic characteristics in 2019–21, 2015–16, and 2005–06, respectively. Column (4) presents the percentage distribution for the pooled sample. The pooled sample includes analytical samples from the fifth, fourth, and third rounds of the National Family and Health Survey. Percentages are adjusted for survey weights.

**Table 2:** Adjusted Odds Ratios (with 95% Confidence Intervals) of different demographic and socioeconomic characteristics on women’s acceptability of PIPV, India.

Characteristics	Acceptability of PIPV			
	NFHS-5	NFHS-4	NFHS-3	Pooled
	(1)	(2)	(3)	(4)
**Women’s Age**				
15–19	Ref			
20–29	1.03	0.96	0.97	0.96 *
	(0.96–1.11)	(0.90–1.03)	(0.91–1.02)	(0.93–1.00)
30–39	1.05	0.95	0.98	0.96 *
	(0.97–1.14)	(0.88–1.02)	(0.92–1.05)	(0.93–1.01)
40–49	1.06	0.97	1.02	0.98
	(0.98–1.16)	(0.89–1.05)	(0.95–1.10)	(0.94–1.03)
**Women’s Education**				
No education	Ref			
Primary	1.05	0.93**	0.92***	0.94***
	(0.99–1.12)	(0.87–0.98)	(0.86–0.98)	(0.91–0.98)
Secondary	0.95 *	0.84***	0.72***	0.81***
	(0.90–1.01)	(0.79–0.89)	(0.67–0.76)	(0.78–0.84)
Higher	0.71***	0.65***	0.38***	0.58***
	(0.64–0.77)	(0.59–0.70)	(0.34–0.41)	(0.55–0.61)
**Marital Status**				
Never married	Ref			
Currently married	1.21***	1.19***	1.06	1.17***
	(1.10–1.34)	(1.09–1.30)	(0.98–1.15)	(1.11–1.23)
Single	1.11	0.96	0.91 *	1
	(0.96–1.29)	(0.85–1.08)	(0.82–1.01)	(0.94–1.08)
**Age at First Cohabitation**				
Less than 18	Ref			
18–21	0.96 *	0.97	0.89***	0.94***
	(0.91–1.00)	(0.92–1.01)	(0.85–0.93)	(0.91–0.96)
More than 21	0.89***	0.90***	0.82***	0.87***
	(0.84–0.95)	(0.84–0.96)	(0.76–0.88)	(0.84–0.91)
**Employment Status**				
Not working	Ref			
Agricultural	1.16***	1.33***	1.11***	1.22***
	(1.09–1.23)	(1.24–1.41)	(1.04–1.18)	(1.18–1.27)
Others	1.05	1.16***	0.98	1.07***
	(0.99–1.11)	(1.10–1.23)	(0.93–1.04)	(1.03–1.10)
Don’t know/missing	1.02	1.52***	2.03**	1.46***
	(0.58–1.78)	(1.25–1.85)	(1.09–3.78)	(1.22–1.76)
**Access to Bank Account**				
No	Ref			
Yes	0.91***	0.92***	0.79***	0.88***
	(0.87–0.96)	(0.88–0.97)	(0.74–0.83)	(0.86–0.91)
**Media Exposure**				
No	Ref			
Yes	0.98	1.02	1	1
	(0.92–1.03)	(0.96–1.09)	(0.93–1.07)	(0.96–1.03)
**Experience of Interparental Violence**				
No	Ref			
Yes	1.75***	1.65***	1.43***	1.63***
	(1.64–1.87)	(1.54–1.76)	(1.34–1.52)	(1.57–1.69)
Don’t know/missing	1.16***	1.07***	1.10***	1.10***
	(1.11–1.21)	(1.03–1.11)	(1.06–1.14)	(1.08–1.13)
**Husband’s Education**				
No education	Ref			
Primary	1.02	1.01	0.95	0.99
	(0.95–1.10)	(0.94–1.08)	(0.89–1.01)	(0.95–1.03)
Secondary	0.94 *	1.01	0.98	0.98
	(0.89–1.01)	(0.95–1.07)	(0.93–1.04)	(0.95–1.02)
Higher	0.92 *	0.95	0.88***	0.91***
	(0.84–1.01)	(0.87–1.03)	(0.80–0.96)	(0.87–0.96)
Don’t know/missing	0.71**	0.94	1	0.94
	(0.53–0.95)	(0.67–1.32)	(0.83–1.22)	(0.80–1.09)
**Religion**				
Hindu	Ref			
Muslim	1.20***	1.22***	1.09 *	1.18***
	(1.10–1.31)	(1.12–1.33)	(0.99–1.20)	(1.12–1.25)
Christian	0.88 *	1.03	1.16**	1.02
	(0.76–1.01)	(0.89–1.19)	(1.00–1.34)	(0.93–1.11)
Others	0.81***	0.92	1.35***	1.03
	(0.71–0.92)	(0.80–1.05)	(1.19–1.54)	(0.95–1.11)
**Social Group**				
UC	Ref			
SC	1.11***	1.10**	1.11***	1.12***
	(1.03–1.20)	(1.01–1.19)	(1.03–1.21)	(1.07–1.17)
ST	0.91 *	0.89**	1.01	0.94 *
	(0.83–1.01)	(0.81–0.98)	(0.90–1.13)	(0.89–1.00)
OBC	0.99	1.01	1.08**	1.04 *
	(0.92–1.05)	(0.94–1.08)	(1.01–1.15)	(1.00–1.08)
Don’t know/missing	0.92	1.24**	1.30***	1.11**
	(0.81–1.06)	(1.05–1.46)	(1.13–1.50)	(1.01–1.20)
**Household Wealth**				
Poorest	Ref			
Poor	0.88***	0.90***	0.94 *	0.92***
	(0.82–0.94)	(0.85–0.97)	(0.87–1.01)	(0.88–0.96)
Middle	0.81***	0.80***	0.91**	0.86***
	(0.75–0.87)	(0.74–0.86)	(0.83–0.99)	(0.82–0.90)
Rich	0.71***	0.70***	0.83***	0.77***
	(0.66–0.78)	(0.64–0.76)	(0.76–0.91)	(0.74–0.81)
Richest	0.54***	0.57***	0.60***	0.58***
	(0.49–0.60)	(0.52–0.63)	(0.54–0.67)	(0.55–0.62)
**Place of Residence**				
Urban	Ref			
Rural	1.23***	1.15***	1.35***	1.25***
	(1.14–1.34)	(1.07–1.24)	(1.24–1.48)	(1.19–1.30)
Survey Round Dummies	No	No	No	Yes
Observations	1,07,281	1,20,238	1,20,956	3,48,475

Notes: The outcome variable is women’s acceptability of PIPV. Columns (1), (2), and (3) represent the adjusted odds of women’s acceptability of PIPV in 2019–21, 2015–16, and 2005–06, respectively. Adjusted odds ratios reported in column (4) represent the adjusted odds of women’s acceptability of PIPV adjusted for the survey years. Estimations are adjusted for the region dummies. Survey round dummies are also included in the pooled sample. Sampling weights provided in the NFHS are used. 95% confidence intervals accounting for cluster survey design are reported in the parentheses. *** p < 0.01, ** p < 0.05, *  p < 0.1.

## Results

### Sample characteristics.

The percentage distribution of demographic and socioeconomic characteristics is presented for each survey round, NFHS-5, NFHS-4, and NFHS-3, in columns (1), (2), and (3) of Table 1, respectively. Column (4) of Table 1 displays the percentage distribution of these characteristics for the pooled sample. In our analytical sample, based on the latest round of NFHS conducted in 2019–21, 16.5% of women are teenagers (aged 15–19), 60.5% are in the early reproductive years (aged 20–39), and 23% are in the late reproductive years (aged 40–49). Around 22.6% of the women haven’t received any education, and the rest have received primary (11.8%), secondary (49.9%), or higher education (15.7%). Women who are currently married (never married) form 72.4% (23%) of the NFHS-5 sample, and 29.9% (28%) entered their first cohabitation at ages less than 18 (18–21), while 14.5% started their first cohabitation at ages 22 and above. Around 69.5% of women are non-working, and the rest are employed in agriculture (14.1%) or other occupations (16.4%). In the NFHS-5 sample, 78.8% of women have access to a bank account, 77.3% are exposed to some form of media, and 12.9% have experienced interparental violence.

In 2019–21, husbands who haven’t received any education form 13.7% of the sample, while the remainder have received primary (11.2%), secondary (40%), or higher education (11.9%). The distribution of household characteristics reveals that 80.8% (14%) of women are Hindu (Muslim), 43.8% (30.8%) are from OBC (SC/ST) backgrounds, 59.4% belong to poor or middle wealth quintiles, and 67.7% live in rural areas. The percentage distribution of these characteristics in the remaining samples aligns with that observed in the NFHS-5 sample.

### Acceptability of PIPV—Patterns and trends.

Fig 2 plots the level of acceptability of PIPV among women aged 15–49 years for the NFHS rounds 3, 4, and 5. It shows a downward trend in the percentage of women who justify PIPV, declining from 55.7% in 2005–06 to 52.4% in 2015–16 and dropping to 45.9% in 2019–21. This decreasing trend in women’s acceptability of PIPV over the years suggests a positive shift towards more progressive attitudes. However, it is important to note that despite this decline, almost half of the women surveyed in 2019–21 still justify PIPV.

Figs 3–6 plot the level of acceptability of PIPV among women by different demographic and socioeconomic characteristics for all three rounds of NFHS. The level of acceptability of PIPV appears notably higher among women aged 30–39 or 40–49 years (47.5% and 49.1% in 2019–21) compared to women aged 15–19 or 20–29 years (43.2% and 43.7% in 2019–21), as depicted in Fig 3a. Nevertheless, a consistent declining trend in the acceptability of PIPV is observed across all age cohorts of women.

Fig 3b illustrates the trends and patterns of acceptability of PIPV among women with different levels of education. With the increase in education levels, the level of acceptability of PIPV decreases, possibly due to more empowerment and the ability to advocate their opinions. Women with higher education exhibit the lowest level of acceptability of PIPV (27.2% in 2005–06; 40.7% in 2015–16; 36.2% in 2019–21), while those with no formal education demonstrate the highest level of acceptability (63.4% in 2005–06; 59.1% in 2015–16; 50.8% in 2019–21). While an overall decline in acceptability is evident over the years, a noteworthy exception is observed among women with higher education, where acceptability initially increased from 27.2% in 2005–06 to 40.7% in 2015–16 before decreasing to 36.2% in 2019–2021.

Fig 3c shows a downward trend in the acceptability of PIPV over time across all the categories of marital status. However, the acceptability of PIPV of married (56.6% in 2005–06; 53.8% in 2015–16; 47.1% in 2019–21) or single (58.3% in 2005–06; 54.2% in 2015–16; 52% in 2019–21) women remain consistently higher than those of unmarried women (51.3% in 2005–06; 47.2% in 2015–16; 41% in 2019–21). This shift in women’s perspectives post-marriage indicates the substantial influence of societal or cultural factors on women’s attitudes in India. The societal stigma surrounding divorce and the perception of marriage as a lifelong commitment may compel women to tolerate violence, justifying it as their husband’s right to punish them.

Additionally, we find a negative gradient between age at first cohabitation and the percentage of women who justify PIPV (Fig 3d). Specifically, the level of acceptability is highest among currently married women who entered their first cohabitation at an age less than 18 years (61% in 2005–06; 56.9% in 2015–16; 49.9% in 2019–21) and lowest among women who initiated their first cohabitation at ages more than 21 (42.3% in 2005–06; 48.3% in 2015–16; 42.5% in 2019–21). This result is plausible as women who marry very young tend to be more naive, less empowered, and, hence, are less opinionated than women who marry later in life [62,80].

According to Fig 4a, women employed in agriculture show higher level of acceptability of PIPV (65.8% in 2005–06; 63.4% in 2015–16; 57% in 2019–21) relative to women who are not working (51.9% in 200–06; 49.6% in 2015–16; 43.2% in 2019–21) or employed in non-agricultural occupations (53.4% in 2005–06; 53.7% in 2015–16; 47.9% in 2019–21). This pattern is interesting to note because it suggests the possibility of a sense of guilt among women who are employed and observed as deviating from traditional gender roles of solely being homemakers [22]. In the Indian context, where women’s engagement in homemaking is considered an enhanced family status [81], those who fail to meet these societal norms might perceive that IPV results from their actions [82], leading to higher acceptability among working women.

Women who have access to a bank account show only marginally lower levels of acceptability of PIPV than women who do not have access (Fig 4b). In the NFHS-5 (2019–21), the level of acceptability among women with access to a bank account (45.9%) is only 0.2 percentage points (p.p.) less than that of women without access (46.1%). It indicates that financial access alone may not be sufficient in changing women’s attitudes towards PIPV. However, women who are exposed to some form of media (such as newspapers, television, or radio) demonstrate notably lower levels of acceptability of PIPV (53.6%, 51.7%, and 45.8%) compared to women with no exposure, whose levels stand at 62.4%, 55.6%, and 46.5% in 2005–06, 2015–16, and 2019–21, respectively (Fig 4c). Moreover, we find a discernible declining trend in the acceptability of PIPV among women, irrespective of their exposure to the media.

In addition to all these factors, women who have witnessed interparental violence internalize their roles as submissive and learn to normalize IPV in their relationships, which shapes their attitudes towards violence victimization [40]. The level of acceptability of PIPV is considerably high among women who have experienced interparental violence, ranging from 67.1% in 2005–06 to 67.6% in 2015–16 and 64.5% in 2019–21 (Fig 5a). Whereas women without any prior exposure to interparental violence show a consistently low and declining level of acceptability (53.4% in 2005–06; 49.3% in 2015–16; 42.6% in 2019–21).

The trends and patterns of women’s acceptability of PIPV by their husbands’ levels of education (Fig 5b) are similar to those observed by their educational levels (Fig 3b). Women whose husbands are uneducated demonstrate the highest levels of acceptability (64.9% in 2005–06; 60.9% in 2015–16; 54.2% in 2019–21), and these levels decline with the increasing levels of husbands’ education. Women married to husbands with higher education show the lowest levels of acceptability of PIPV (36.1% in 2005–06; 42.7% in 2015–16; 38.2% in 2019–21). Women married to highly educated husbands may have higher education levels and be part of social circles that embrace egalitarian values. Hence, these women might exhibit more awareness and progressive attitudes towards PIPV.

Over 15 years, there has been a clear decline in women’s acceptability of PIPV across various household-level characteristics such as religion (Fig 6a), social group (Fig 6b), and wealth index (Fig 6c), indicating a more progressive shift in women’s attitudes over time. However, women following Muslim (46.8% in 2019–21) or Christian (54% in 2019–21) religions compared to Hindu religion (46.1% in 2019–21) and women belonging to socially disadvantaged classes and tribal communities (49.1%, 45%, 48.9% among SC, ST, and OBC women in 2019–21) compared to UC (38.1% in 2019–21) demonstrate higher levels of acceptability of PIPV. Women from the poorest households show the highest level of acceptability to PIPV at 63.7% in 2005–06, which reduced to about 57.5% in 2015–16 and 48.1% in 2019–21, though this level is still higher than the level in richest households (35.2%) by 12.9 p.p. in 2019–21. In contrast, women in the richest households have the lowest levels of acceptability of PIPV at 37.9%, 41.3%, and 35.2% in 2005–06, 2015–16, and 2019–21, respectively. Women from these marginalized communities have limited access to strong means of livelihood and fear the consequences of marriage dissolution. Hence, these women are conditioned to abide by the patriarchal norms and justify PIPV for secured sustenance of themselves and their children [83,84].

Urbanization often correlates to modernization and better access to information and opportunities, which may, in turn, also influence women’s attitudes to challenge traditional gender beliefs. Fig 6d illustrates that women from urban regions (45.2% in 2005–06, 47.4% in 2015–16, and 41% in 2019–21) exhibit lower levels of acceptability of PIPV compared to women from rural regions. The level of acceptability among rural women was as high as 60.8% in 2005–06, which has shown a gradual decline to 55.2 in 2015–16, ultimately reaching 48.3% in 2019–21.

Fig 7 highlights significant regional variations in women’s acceptability of PIPV across India, with notable trends observed between 2005–06 and 2019–21. In the NFHS-3 (2005–06), the highest levels of acceptability are concentrated in the Northeastern states, including Manipur (90.5%), Mizoram (84.2%), Nagaland (81.8%), Sikkim (76.9%), and Arunachal Pradesh (73.6%). Similarly, elevated levels are observed in the South Indian states of Andhra Pradesh (77.8%), Tamil Nadu (68%), Karnataka (67.9%), and Kerala (67.2%). In contrast, the lowest levels of acceptability during this period are reported in Himachal Pradesh (28.9), Delhi (33.1), Chhattisgarh (33.9), and Goa (40.7).

Over the subsequent 15 years, the largest declines in the acceptability of PIPV have occurred in the Northeastern states. For instance, Sikkim has shown a reduction from 76.9% to 33.5%, Arunachal Pradesh from 73.6% to 34.5%, Nagaland from 81.8% to 25%, and Mizoram from 84.2% to 35%. These substantial declines suggest a transformation in societal attitudes, potentially driven by improvements in education, awareness campaigns, and exposure to more egalitarian norms. Conversely, certain states such as Uttar Pradesh, Maharashtra, Goa, and Chhattisgarh show only marginal declines in acceptability levels over this period. Notably, West Bengal exhibits stagnation, with the level of acceptability remaining unchanged at 42.7%.

In South India, however, a reverse trend is observed. Rather than declining, women’s acceptability of PIPV has increased in Andhra Pradesh (77.8% to 84.6%), Karnataka (67.9% to 77.4%), and Tamil Nadu (68% to 78.7%) between 2005–06 and 2019–21. In the NFHS-5 (2019–21), these states, along with Telangana (84.7%), report the highest levels of PIPV acceptability. Evidence suggests that Southern India has a more egalitarian mindset because of the prevalence of cross-cousin marriages, the absence of the purdah system, and better infrastructure for women empowerment [85,86]. Despite these anti-patriarchal practices, unspoken traditional societal norms might be operating in Southern India [87,88]. Women defying these norms may justify wife-beating as a punishment for transgressing from their roles of being good mothers, wives, or daughters-in-law.

### Acceptability of PIPV—Determinants.

The adjusted odds ratios (AOR) and their 95% confidence intervals (CI) from the logistic regression analysis between demographic and socioeconomic characteristics and women’s likelihood of accepting PIPV in India for each round are reported in columns (1), (2), and (3) of Table 2. Adjusted odds ratios are also estimated separately for the pooled sample and presented in column (4) of Table 2.

In the latest round (2019–21) of the NFHS, women’s increasing levels of education, age at first cohabitation, access to bank accounts, and higher levels of spousal education show significant negative associations with women’s acceptability of PIPV. Women with higher education (AOR 0.71; 95% CI, 0.64–0.77) are less likely to justify PIPV than those without education. Women who initiate their first cohabitation after 21 years (AOR 0.89; 95% CI, 0.84–0.95) are at lesser odds of having acceptability to PIPV than women who enter their first cohabitation at a relatively young age of less than 18 years. Similarly, women with some form of financial access, such as bank accounts, have lesser odds (AOR 0.91; 95% CI; 0.87–0.96) of accepting PIPV than their counterparts. These findings confirm how women’s exposure to more egalitarian networks via schooling or increased financial access may change their behaviors learned from traditional norms and may actually make them challenge patriarchal attitudes. Hence, these women hold more egalitarian beliefs and are less likely to tolerate PIPV.

Contrary to these trends, women’s marital status, employment status, and exposure to interparental violence are associated with women’s increased acceptability of PIPV. Married women are more likely to show attitudes justifying PIPV (AOR 1.21; 95% CI; 1.10–1.34) than never-married women. Women employed in agriculture (AOR 1.16; 95% CI, 1.09–1.23) are more likely to justify PIPV than women who are not working. These findings align with the normalization theory, which provides a critical lens for understanding women’s behaviors within the Indian context, where divorce or separation carries significant stigma. In such settings, IPV is often perceived as an inherent aspect of marital life, leading many married women to accept this reality to preserve their marriages willingly. Similarly, women employed in agriculture are more likely to internalize blame for failing to fulfill traditional household duties, thereby perceiving IPV as a consequence of their actions and accepting it as justified. On the other hand, women who have witnessed their mothers being beaten by their fathers are significantly more likely to internalize their roles as victims of violence, showing a greater likelihood of justifying PIPV (AOR 1.75; 95% CI, 1.64–1.87) compared to those without exposure to interparental violence.

Furthermore, household-level characteristics, such as wealth, show that women from wealthier households are (AOR 0.54; 95% CI; 0.49–0.60 for richest, AOR 0.71; 95% CI; 0.66–0.78 for rich, AOR 0.81; 95% CI; 0.75–0.87 for middle and AOR 0.88; 95% CI; 0.82–0.94 for poor) less likely to justify PIPV compared to women from the poorest households. Moreover, the AOR shows a declining likelihood of justification with increasing household wealth: AOR 0.88 (95% CI, 0.82–0.94) for poor households, AOR 0.81 (95% CI, 0.75–0.87) for middle-income households, AOR 0.71 (95% CI, 0.66–0.78) for rich households, and AOR 0.54 (95% CI, 0.49–0.60) for the richest households. This trend may be attributed to increased financial stability and reduced stress-induced conflict within wealthier households. The logistic regression results find that Muslim women, with an AOR of 1.20 (95% CI; 1.10–1.31), are more likely to justify PIPV than women following the Hindu religion. However, women from other religions (AOR 0.81; 95% CI, 0.71–0.92) show a lower likelihood of acceptability of PIPV compared to women from the Hindu religion. Social minority groups and place of residence show significant positive associations with women’s attitudes towards PIPV. Women from SC (AOR 1.11; 95% CI, 1.03–1.20) backgrounds exhibit a greater likelihood of the acceptability of PIPV than women belonging to UC backgrounds. Similarly, women residing in rural areas are (AOR 1.23; 95% CI; 1.14–1.34) more likely to justify PIPV than women in urban areas, attributing to the less developed and more conservative nature of rural societies in India.

The results based on the other samples are qualitatively similar to those for the NFHS-5 sample except for religion. Analysis based on the NFHS-3 sample in column 3 shows that women following the Christian religion (AOR 1.16; 95% CI, 1.00–1.34) or other religions (AOR 1.35; 95% CI, 1.19–1.54) show a higher likelihood of acceptability of PIPV compared to women following the Hindu religion.

## Limitations

There are two major limitations of this study. First, there could be reporting errors in the outcome variable, women’s acceptability of PIPV. Schuler et al. (2012), show the underrepresentation of individuals justifying IPV in Bangladesh’s demographic and health survey data [39]. Participants in face-to-face interviews may tend to underreport or misreport their attitudes toward IPV due to social desirability bias pertaining to the sensitive nature of these questions [89]. Second, while we tried to control for a host of explanatory variables in the logistic regressions, we cannot definitively say that we have been able to control for all the factors affecting women’s attitudes towards PIPV. Consequently, the regression findings cannot be used to make causal inferences.

## Discussion

In predominantly traditional patriarchal societies like India, women often find themselves in a subordinate role, referred to as the ‘second gender,’ where societal expectations dictate obedience to fathers, husbands, and, later, sons. According to social learning theory [24,25], the acceptability of PIPV becomes ingrained as a norm persisting across generations, seamlessly becoming a part of daily life in such societies. Consequently, women within these social structures are more susceptible to IPV [8,9,90]. It is imperative to estimate the levels and determinants of acceptability of PIPV among women to identify the most vulnerable groups and formulate targeted policy interventions for this population.

This paper finds evidence of a declining trend in the acceptability of PIPV among Indian women over the past 15 years. Despite this declining trend, around 46% of women in India continue to justify PIPV based on instances where they perceive deviation from the traditional gender norms and societal expectations. Our findings reveal that the level of acceptability of PIPV is most pronounced among women belonging to demographically and socioeconomically disadvantaged backgrounds. These include women characterized by a lack of formal education, early marriage, no exposure to media, past exposure to interparental violence, affiliation with religious minorities, backward castes, tribal communities or grappled with poverty, and predominantly residing in rural areas.

We find that a range of factors is strongly associated with women’s acceptability of PIPV. Higher levels of women’s own education and spousal education are negatively associated with the likelihood of justifying PIPV. This is consistent with previous research conducted in different settings, including Bangladesh [91], Ghana [33], Nigeria [84], Papua New Guinea [89], and Zimbabwe [36]. It suggests that education not only acts as a vehicle for empowerment [92] but also plays a pivotal role in reshaping women’s perceptions [63]. Due to their knowledge and exposure, women with higher education tend to perceive PIPV as a negative phenomenon and, thus, are less likely to justify it [33]. Corroborating with the previous findings, we find a strong negative gradient between household wealth and odds for justification of PIPV among women, suggesting progressive attitudes with increasing household economic status foster an environment conducive to gender equality [33,34,84,91].

Women exposed to interparental violence are more likely to justify PIPV than their counterparts. It is plausible due to the transmission of norms they have witnessed for generations, where their mothers were physically assaulted by their fathers, and they have grown up believing it to be a normal part of married life. Given that a large proportion of Indian women work in the informal sector or occupy low-paid jobs marked by exploitative conditions [93], women might encounter similar patriarchal social structures within the workplace and reinforce the idea of male superiority among them [19]. Hence, it is not surprising to find that women engaged in employment have a higher likelihood of justifying PIPV. In line with the literature [36], residence in rural areas significantly predicts regressive attitudes towards PIPV.

These findings underscore the need for policy interventions tailored to meet the specific needs of the most vulnerable groups of women, particularly those who are illiterate, married at young ages, and situated at an intersection of poverty, religious or social minorities, and rural residence. Our study highlights the importance of intervention schemes aimed at increasing the education levels of both men and women (e.g., the Mid-day Meal Scheme, Right to Education Act, etc.), increasing women’s age at marriage (e.g., Kanyashree Prakalpa, etc.), and fostering socioeconomic equity. Most importantly, awareness programs promoting egalitarian gender norms and discouraging patriarchal gender beliefs must be designed, particularly for women from marginalized communities. Implementing such measures is essential for further reducing the acceptability of PIPV and addressing the persistent challenges observed in these vulnerable groups.

## Supporting information

S1 Appendix Women's exposure to physical intimate partner violence, sexual intimate partner violence, and emotional intimate partner violence.
(DOCX)

## References

[1] WHO. Violence against women 2021. Available from: https://www.who.int/news-room/fact-sheets/detail/violence-against-women

[2] Breiding M, Basile KC, Smith SG, Black MC, Mahendra RR. Intimate partner violence surveillance: uniform definitions and recommended data elements. Version 2.0. 2015. Available from: https://stacks.cdc.gov/view/cdc/31292

[3] Sardinha L, Maheu-Giroux M, Stöckl H, Meyer SR, García-Moreno C. Global, regional, and national prevalence estimates of physical or sexual, or both, intimate partner violence against women in 2018. Lancet. 2022;399(10327):803–13. doi: 10.1016/S0140-6736(21)02664-7 35182472 PMC8885817

[4] Campbell JC. Health consequences of intimate partner violence. Lancet. 2002;359(9314):1331–6. doi: 10.1016/S0140-6736(02)08336-8 11965295

[5] Lindhorst T, Oxford M, Gillmore MR. Longitudinal effects of domestic violence on employment and welfare outcomes. J Interpers Violence. 2007;22(7):812–28. doi: 10.1177/0886260507301477 17575064 PMC1952653

[6] Aizer A. Poverty, violence, and health. J Human Resources. 2011;46(3):518–38. doi: 10.3368/jhr.46.3.518 PMC4019993 24839303

[7] Ackerson LK, Subramanian SV. Domestic violence and chronic malnutrition among women and children in India. Am J Epidemiol. 2008;167(10):1188–96. doi: 10.1093/aje/kwn049 18367471 PMC2789268

[8] Mookerjee M, Ojha M, Roy S. Spousal beliefs and intimate partner violence: are we conditioned to internalize patriarchal norms? Economics Letters. 2021;202:109811. doi: 10.1016/j.econlet.2021.109811

[9] Dasgupta S. Attitudes about wife-beating and incidence of domestic violence in India: an instrumental variables analysis. J Fam Econ Iss. 2019;40(4):647–57. doi: 10.1007/s10834-019-09630-6

[10] Rani M, Bonu S. Attitudes toward wife beating: a cross-country study in Asia. J Interpers Violence. 2009;24(8):1371–97. doi: 10.1177/0886260508322182 18718881

[11] Visaria L. Violence against women: a field study. Economic and Political Weekly. 2000. p. 1742–51. Available from: https://www.jstor.org/stable/4409296

[12] IIPS, ICF. National Family Health Survey (NFHS-5), 2019-21. Mumbai, India: International Institute for Population Sciences (IIPS); 2022. Available from: https://www.iipsdata.ac.in/datacatalog_detail/1

[13] Bhattacharya H. Mass media exposure and attitude towards spousal violence in India. Soc Sci J. 2016;53(4):398–416. doi: 10.1016/j.soscij.2016.02.011

[14] Hayes BE, Boyd KA. Influence of individual- and national-level factors on attitudes toward intimate partner violence. Sociol Perspect. 2016;60(4):685–701. doi: 10.1177/0731121416662028

[15] Serrano-Montilla C, Lozano LM, Bender M, Padilla J-L. Individual and societal risk factors of attitudes justifying intimate partner violence against women: a multilevel cross-sectional study. BMJ Open. 2020;10(12):e037993. doi: 10.1136/bmjopen-2020-037993 33303434 PMC7733202

[16] Chattopadhyay S, Sidharth J. Gender norms, domestic violence, and the southern Indian puzzle. Econ Polit Wkly. 2022;57:1–14. Available from: https://www.epw.in/engage/article/gender-norms-domestic-violence-and-southern-indian

[17] Dev P, Vijayalakshmi A, Unni J. Does a man’s hardship matter more than a woman’s? Reasons for justifying domestic violence. Econ Polit Wkly. 2023;58:60–8. Available from: https://www.epw.in/journal/2023/40/special-articles/does-mans-hardship-matter-morewomans.html

[18] Jejeebhoy SJ. Wife-beating in rural India: a husband’s right? Evidence from survey data. Econ Polit Wkly. 1998; p. 855–62. Available from: https://www.jstor.org/stable/4406642.

[19] Rani M, Bonu S, Diop-Sidibe N. An empirical investigation of attitudes towards wife-beating among men and women in seven sub-Saharan African countries. Afr J Reprod Health. 2004;8(3):116–36. 17348330

[20] Ackerson LK, Subramanian SV. State gender inequality, socioeconomic status and Intimate Partner Violence (IPV) in India: a multilevel analysis. Aust J Social Issues. 2008;43(1):81–102. doi: 10.1002/j.1839-4655.2008.tb00091.x

[21] Heise L, Ellsberg M, Gottemoeller M. Ending violence against women (population reports, Series L, No. 11). Baltimore: Johns Hopkins University School of Public Health, Center for Communications Programs; 1999. Available from: https://xyonline.net/sites/xyonline.net/files/Population%20Reports%2C%20Ending%20Violence%20Against%20Women%2099_0.pdf

[22] Dhanaraj S, Mahambare V. Male backlash and female guilt: women’s employment and intimate partner violence in Urban India. Feminist Economics. 2021;28(1):170–98. doi: 10.1080/13545701.2021.1986226

[23] Krishnan S. Gender, caste, and economic inequalities and marital violence in rural South India. Health Care Women Int. 2005;26(1):87–99. doi: 10.1080/07399330490493368 15764463

[24] Bandura A, Walters RH. Social learning and personality development. 1963. Available from: https://psycnet.apa.org/record/1963-35030-000

[25] Bandura A, Park R. Recent trends in social learning theory. New. 1972. doi: 10.1016/B978-0-12-545050-8.50008-0

[26] Heise LL. Violence against women: an integrated, ecological framework. Violence Against Women. 1998;4(3):262–90. doi: 10.1177/1077801298004003002 12296014

[27] Uthman OA, Lawoko S, Moradi T. Factors associated with attitudes towards intimate partner violence against women: a comparative analysis of 17 sub-Saharan countries. BMC Int Health Hum Rights. 2009;9:14. doi: 10.1186/1472-698X-9-14 19619299 PMC2718859

[28] Lundgren E, Heimer G, Westerstrand J, Kalliokoski A-M. Mens violence against women in “equal” Sweden-a prevalence study. Brottsoffermyndigheten and Uppsala Universitet Sweden 2001. Available from: https://www.uu.se/download/18.75e37daa18e79b469f4324de/1712427056282/captured-queen_eng.pdf

[29] Okenwa-Emegwa L, Lawoko S, Jansson B. Attitudes toward physical intimate partner violence against women in Nigeria. Sage Open. 2016;6(4). doi: 10.1177/2158244016667993

[30] Sayem AM, Begum HA, Moneesha SS. Attitudes towards justifying intimate partner violence among married women in Bangladesh. J Biosoc Sci. 2012;44(6):641–60. doi: 10.1017/S0021932012000223 22687269

[31] Allen CM, Straus MA. Resources, power, and husband-wife violence. The Social Causes of Husband-Wife Violence. 1980; p. 188–208. Available from: https://www.ojp.gov/ncjrs/virtual-library/abstracts/resources-power-and-husband-wife-violence.

[32] Cools S, Kotsadam A. Resources and intimate partner violence in Sub-Saharan Africa. World Develop. 2017;95:211–30. doi: 10.1016/j.worlddev.2017.02.027

[33] Adu C. Socio-economic inequalities in intimate partner violence justification among women in Ghana: analysis of the 2014 Ghana Demographic and Health Survey data. Int Health. 2023;15(2):182–8. doi: 10.1093/inthealth/ihac032 35640232 PMC9977217

[34] Doku DT, Asante KO. Women’s approval of domestic physical violence against wives: analysis of the Ghana demographic and health survey. BMC Womens Health. 2015;15 120. doi: 10.1186/s12905-015-0276-0 26691763 PMC4687112

[35] Lawoko S. Predictors of attitudes toward intimate partner violence: a comparative study of men in Zambia and Kenya. J Interpers Violence. 2008;23(8):1056–74. doi: 10.1177/0886260507313972 18292405

[36] Hindin MJ. Understanding women’s attitudes towards wife beating in Zimbabwe. Bull World Health Organ. 2003;81(7):501–8. 12973642 PMC2572507

[37] Tran TD, Nguyen H, Fisher J. Attitudes towards intimate partner violence against women among women and men in 39 low- and middle-income countries. PLoS One. 2016;11(11):e0167438. doi: 10.1371/journal.pone.0167438 27893861 PMC5125706

[38] Bates LM, Ruth S, Islam F, Islam MK. Factores socioeconómicos y procesos relacionados con la violencia doméstica en zonas rurales de Bangladesh. Perspectiva de Int Fam Plan. 2004;30:190–9. Available from: https://www.researchgate.net/profile/Khairul-Islam/publication/242552075_Factores_socioeconomicos_y_procesos_relacionados_con_la_violencia_domestica_en_zonas_rurales_de_Bangladesh/links/00b49537435ae60e0d000000/Factores-socioeconomicos-y-procesos-relacionados-con-la-violencia-domestica-en-zonas-rurales-de-Bangladesh.pdf.

[39] Schuler SR, Yount KM, Lenzi R. Justification of wife beating in rural Bangladesh: a qualitative analysis of gender differences in responses to survey questions. Violence Against Women. 2012;18(10):1177–91. doi: 10.1177/1077801212465152 23136180 PMC3721193

[40] Jewkes R, Levin J, Penn-Kekana L. Risk factors for domestic violence: findings from a South African cross-sectional study. Soc Sci Med. 2002;55(9):1603–17. doi: 10.1016/s0277-9536(01)00294-5 12297246

[41] Jensen R, Oster E. The Power of TV: cable television and women’s status in India*. Quart J Econ. 2009;124(3):1057–94. doi: 10.1162/qjec.2009.124.3.1057

[42] Yount KM, Li L. Women’s “Justification” of domestic violence in Egypt. J of Marriage and Family. 2009;71(5):1125–40. doi: 10.1111/j.1741-3737.2009.00659.x

[43] Behrman J, Frye M. Attitudes toward intimate partner violence in dyadic perspective: evidence from Sub-Saharan Africa. Demography. 2021;58(3):1143–70. doi: 10.1215/00703370-9115955 33835134 PMC10768745

[44] Hindin MJ, Adair LS. Who’s at risk? Factors associated with intimate partner violence in the Philippines. Soc Sci Med. 2002;55(8):1385–99. doi: 10.1016/s0277-9536(01)00273-8 12231016

[45] Antai D, Antai J. Collective violence and attitudes of women toward intimate partner violence: Evidence from the Niger Delta. BMC Int Health Hum Rights. 2009;9:12. doi: 10.1186/1472-698X-9-12 19508708 PMC2702345

[46] Boyle MH, Georgiades K, Cullen J, Racine Y. Community influences on intimate partner violence in India: Women’s education, attitudes towards mistreatment and standards of living. Soc Sci Med. 2009;69(5):691–7. doi: 10.1016/j.socscimed.2009.06.039 19619925

[47] Vanderende KE, Yount KM, Dynes MM, Sibley LM. Community-level correlates of intimate partner violence against women globally: a systematic review. Soc Sci Med. 2012;75(7):1143–55. doi: 10.1016/j.socscimed.2012.05.027 22762950

[48] Yakubovich AR, Stöckl H, Murray J, Melendez-Torres GJ, Steinert JI, Glavin CEY, et al. Prospective risk and protective factors for intimate partner violence victimisation among women: a systematic review and meta-analysis. Lancet. 2017;390:S13. doi: 10.1016/s0140-6736(17)32948-3 PMC5993370 29771615

[49] Aizer A. The gender wage gap and domestic violence. Am Econ Rev. 2010;100(4):1847–59. doi: 10.1257/aer.100.4.1847 25110354 PMC4123456

[50] Tertilt M, van den Berg GJ. The association between own unemployment and violence victimization among female youths. Jahrbücher Für Nationalökonomie Und Statistik 2015;235:499–516.

[51] Anderberg D, Rainer H, Wadsworth J, Wilson T. Unemployment and domestic violence: theory and evidence. Econ J. 2015;126(597):1947–79. doi: 10.1111/ecoj.12246

[52] Li T, Pandya S, Sekhri S. Repelling rape: foreign direct investment empowers women. unpublished manuscript 2020. Available from: https://projects.iq.harvard.edu/files/pegroup/files/pandya_et_al_5.19.pdf

[53] Bhalotra S, Kambhampati U, Rawlings S, Siddique Z. Intimate partner violence: the influence of job opportunities for men and women. World Bank Econ Rev. 2019;35(2):461–79. doi: 10.1093/wber/lhz030

[54] Guarnieri E, Rainer H. Colonialism and female empowerment: a two-sided legacy. J Dev Econ. 2021;151:102666. doi: 10.1016/j.jdeveco.2021.102666

[55] Tur-Prats A. Unemployment and intimate partner violence: a cultural approach. J Econ Behav Organization. 2021;185:27–49. doi: 10.1016/j.jebo.2021.02.006

[56] Tur-Prats A. Family types and intimate partner violence: a historical perspective. Rev Econ Statist. 2019;101(5):878–91. doi: 10.1162/rest_a_00784

[57] González L, Rodríguez-Planas N. Gender norms and intimate partner violence. J Econ Behav Organization. 2020;178:223–48. doi: 10.1016/j.jebo.2020.07.024

[58] Alesina A, Brioschi B, La Ferrara E. Violence against women: a cross‐cultural analysis for Africa. Economica. 2020;88(349):70–104. doi: 10.1111/ecca.12343

[59] Erten B, Keskin P. For better or for worse?: education and the prevalence of domestic violence in Turkey. Am Econ J Appl Econ. 2018;10(1):64–105. doi: 10.1257/app.20160278

[60] Papageorge NW, Pauley GC, Cohen M, Wilson TE, Hamilton BH, Pollak RA. Health, human capital and domestic violence. J Hum Resour. 2021;56(4):997–1030. doi: 10.3368/jhr.56.4.1115-7543r5 35321345 PMC8939878

[61] Amaral S, Bhalotra SR. Population sex ratios and violence against women: The long-run effects of sex selection in India. ISER Working Paper Series; 2017. Available from: https://hdl.handle.net/10419/200361

[62] Roychowdhury P, Dhamija G. The causal impact of women’s age at marriage on domestic violence in India. Fem Econ. 2021;27(3):188–220. doi: 10.1080/13545701.2021.1910721

[63] Roychowdhury P, Dhamija G. Don’t cross the line: bounding the causal effect of hypergamy violation on domestic violence in India. J R Stat Soc Ser A Stat Soc. 2022;185(4):1952–78. doi: 10.1111/rssa.12858

[64] Ojha M, Babbar K. Power to choose? Examining the link between contraceptive use decision and domestic violence. Econ Hum Biol. 2024;55:101416. doi: 10.1016/j.ehb.2024.101416 39154411

[65] Stevenson B, Wolfers J. Bargaining in the shadow of the law: divorce laws and family distress. Quart J Econ. 2006;121(1):267–88. doi: 10.1093/qje/121.1.267

[66] García-Ramos A. Divorce laws and intimate partner violence: evidence from Mexico. J Dev Econ. 2021;150:102623. doi: 10.1016/j.jdeveco.2020.102623

[67] Tauchen HV, Witte AD, Long SK. Domestic violence: a nonrandom affair. Int Econ Rev. 1991; 491–511. doi: 10.2307/2526888

[68] Card D, Dahl GB. Family violence and football: the effect of unexpected emotional cues on violent behavior. Q J Econ. 2011;126(1):103–43. doi: 10.1093/qje/qjr001 21853617 PMC3712874

[69] Bloch F, Rao V. Terror as a bargaining instrument: a case study of dowry violence in Rural India. Am Econ Rev. 2002;92(4):1029–43. doi: 10.1257/00028280260344588

[70] Eswaran M, Malhotra N. Domestic violence and women’s autonomy in developing countries: theory and evidence. Canadian J Econ. 2011;44(4):1222–63. doi: 10.1111/j.1540-5982.2011.01673.x

[71] Anderberg D, Rainer H. Economic abuse: a theory of intrahousehold sabotage. J Public Econ. 2013;97:282–95. doi: 10.1016/j.jpubeco.2012.10.008

[72] Iyengar R. Does the certainty of arrest reduce domestic violence? Evidence from mandatory and recommended arrest laws. J Public Econ. 2009;93(1–2):85–98. doi: 10.1016/j.jpubeco.2008.09.006

[73] Aizer A, Bó PD. Love, hate and murder: commitment devices in violent relationships. J Public Econ. 2009;93(3–4):412–28. doi: 10.1016/j.jpubeco.2008.09.011 24244055 PMC3826260

[74] Angelucci M. Love on the rocks: domestic violence and alcohol abuse in Rural Mexico. BE J Econ Anal Policy. 2008;8(1). doi: 10.2202/1935-1682.1766

[75] Bobonis GJ, González-Brenes M, Castro R. Public Transfers and Domestic Violence: The Roles of Private Information and Spousal Control. American Economic Journal: Economic Policy. 2013;5(1):179–205. doi: 10.1257/pol.5.1.179

[76] Hidrobo M, Fernald L. Cash transfers and domestic violence. J Health Econ. 2013;32(1):304–19. doi: 10.1016/j.jhealeco.2012.11.002 23237793

[77] Dasgupta K, Pacheco G. The impact of child welfare legislation on domestic violence-related homicide rates. Health Econ. 2018;27(5):908–15. doi: 10.1002/hec.3643 29464788

[78] IIPS, ICF. National Family Health Survey (NFHS-3), 2005-06. Mumbai, India: International Institute for Population Sciences (IIPS); 2007. Available from: https://www.iipsdata.ac.in/datacatalog_detail/1

[79] IIPS, ICF. National Family Health Survey (NFHS-4), 2015-16. Mumbai, India: International Institute for Population Sciences (IIPS); 2017. Available from: https://www.iipsdata.ac.in/datacatalog_detail/1

[80] Field E, Ambrus A. Early marriage, age of menarche, and female schooling attainment in Bangladesh. J Polit Econ. 2008;116(5):881–930. doi: 10.1086/593333

[81] Carswell G. Struggles over work take place at home: women’s decisions, choices and constraints in the Tiruppur textile industry, India. Geoforum. 2016;77:134–45. doi: 10.1016/j.geoforum.2016.10.009

[82] Overstreet NM, Quinn DM. The intimate partner violence stigmatization model and barriers to help seeking. Social psychological perspectives on stigma, Routledge; 2016, p. 109–22. Available from: https://www.taylorfrancis.com/chapters/edit/10.4324/9781315540696-12/intimate-partner-violence-stigmatization-model-barriers-help-seeking-nicole-overstreet-diane-quinn 10.1080/01973533.2012.746599 PMC3601798 23524454

[83] Haj-Yahia MM. The incidence of wife abuse and battering and some sociodemographic correlates as revealed by two national surveys in Palestinian society. J Fam Viol. 2000;15:347–74. doi: 10.1023/a:1007554229592

[84] Okenwa-Emegwa L, Lawoko S, Jansson B. Attitudes toward physical intimate partner violence against women in Nigeria. Sage Open. 2016;6(4). doi: 10.1177/2158244016667993

[85] Dyson T, Moore M. On Kinship structure, female autonomy, and demographic behavior in India. Popul Dev Rev. 1983;9(1):35. doi: 10.2307/1972894

[86] Rahman L, Rao V. The determinants of gender equity in India: examining Dyson and Moore’s thesis with New Data. Popul Dev Rev. 2004;30(2):239–68. doi: 10.1111/j.1728-4457.2004.012_1.x

[87] Panchanadeswaran S, Johnson SC, Go VF, Srikrishnan AK, Sivaram S, Solomon S, et al. Using the theory of gender and power to examine experiences of partner violence, sexual negotiation, and risk of HIV/AIDS among economically disadvantaged women in Southern India. J Aggression Maltreat Trauma. 2007;15(3–4):155–78. doi: 10.1080/10926770802097327

[88] Kundapur R, Shetty SM, Kempaller VJ, Kumar A, Anurupa M. Violence against educated women by intimate partners in Urban Karnataka, India. Indian J Community Med. 2017;42(3):147–50. doi: 10.4103/ijcm.IJCM_41_16 28852277 PMC5561691

[89] Adu C, Asare BY-A, Agyemang-Duah W, Adomako EB, Agyekum AK, Peprah P. Impact of socio-demographic and economic factors on intimate partner violence justification among women in union in Papua New Guinea. Arch Public Health. 2022;80(1):136. doi: 10.1186/s13690-022-00889-0 35551645 PMC9097318

[90] Jewkes R. Intimate partner violence: causes and prevention. Lancet. 2002;359(9315):1423–9. doi: 10.1016/S0140-6736(02)08357-5 11978358

[91] Biswas RK, Rahman N, Kabir E, Raihan F. Women’s opinion on the justification of physical spousal violence: a quantitative approach to model the most vulnerable households in Bangladesh. PLoS One. 2017;12(11):e0187884. doi: 10.1371/journal.pone.0187884 29161277 PMC5697832

[92] Le K, Nguyen M. How education empowers women in developing countries. BE J Econ Anal Policy. 2020;21(2):511–36. doi: 10.1515/bejeap-2020-0046

[93] Mondal B, Ghosh J, Chakraborty S, Mitra S. Women Workers in India: Labour Force Trends, Occupatonal Diversificaton and Wage Gaps 2018. Available from: http://publications.azimpremjiuniversity.edu.in/id/eprint/4334.

