# Supplementary material for: Women’s attitudes towards physical intimate partner violence in India: Trends, patterns, and determinants
Source: PLoS One. 2025 Mar 12;20(3):e0318350. doi: 10.1371/journal.pone.0318350 (PMC12051491; doi:10.1371/journal.pone.0318350)

**Women's Attitudes Towards Physical Intimate Partner Violence in India: Trends, Patterns, and Determinants**

**Appendix**

In order to estimate the trends and patterns of women's exposure to physical intimate partner violence (PIPV), sexual intimate partner violence (SIPV), and emotional intimate partner violence (EIPV) in the last 12 months, our analytical sample consists of 64339, 62219, and 60089 currently married women from rounds 3, 4, and 5 who are selected for the state module and have complete data on the intimate partner violence, demographic and socioeconomic characteristics. We have used the domestic violence sample weights to estimate the trend and patterns of the women's exposure to PIPV, SIPV, and EIPV.

**Fig A1. Women**'**s exposure to PIPV in 2005-06, 2015-16, and 2019-21, India.**

**Fig A2. Women**'**s exposure to PIPV by their age, education, and age at first cohabitation in 2005-06, 2015-16, and 2019-21, India.**

(a) (b)

(c)

**Fig A3. Women**'**s exposure to PIPV by their employment status, access to bank account, and media exposure in 2005-06, 2015-16, and 2019-21, India.**

(a) (b)

(c)

**Fig A4. Women**'**s exposure to PIPV by their experience of interparental violence and husband’s education in 2005-06, 2015-16, and 2019-21, India.**

(a) (b)

**Fig A5. Women**'**s exposure to PIPV by religion, social group, household wealth, and place of residence in 2005-06, 2015-16, and 2019-21, India.**

(a) (b)

(c) (d)

**Fig A6. Women**'**s exposure to PIPV by different states and union territories, in 2005-06, 2015-16, and 2019-21, India.**


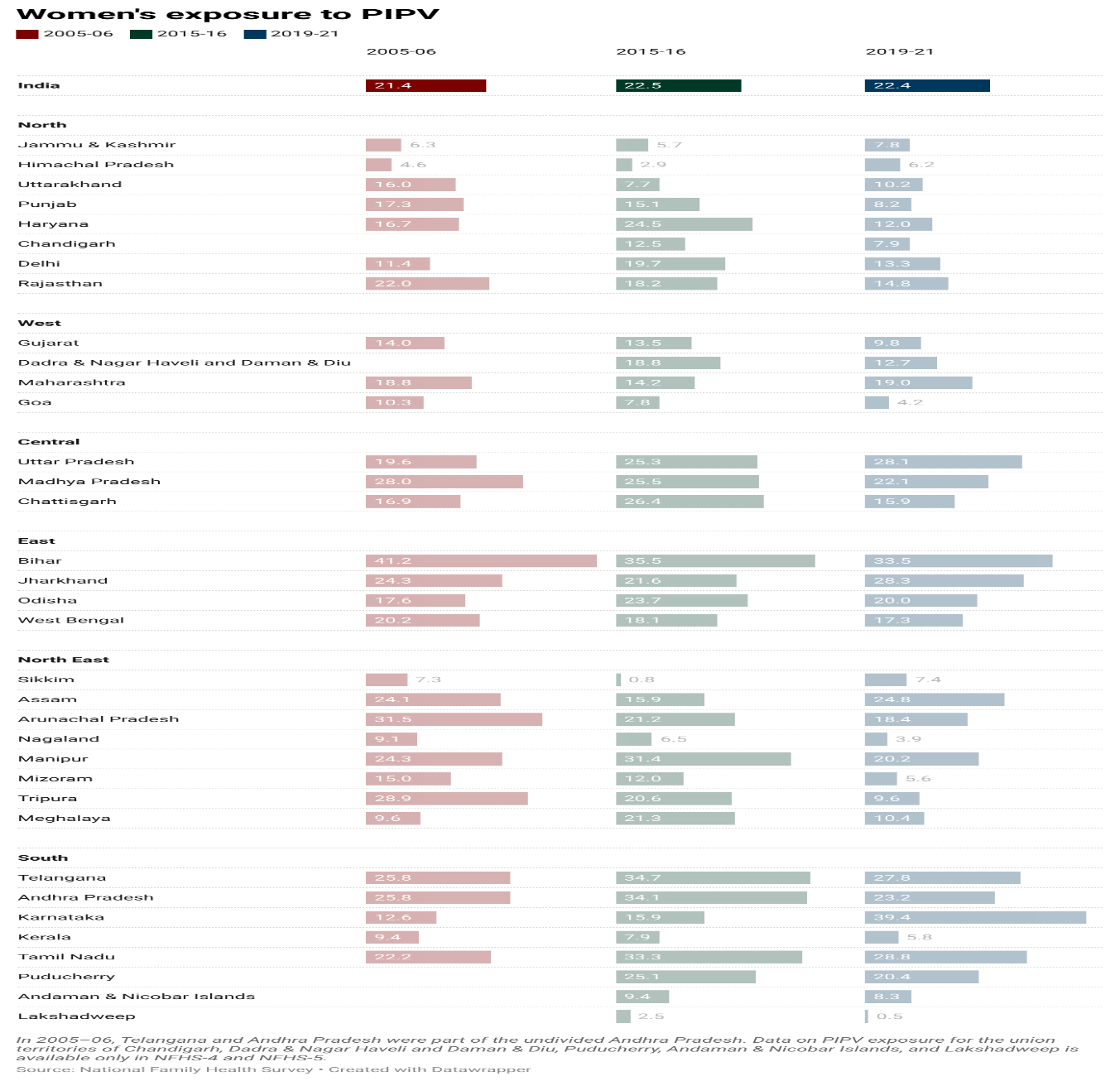


**Fig A7. Women**'**s exposure to SIPV in 2005-06, 2015-16, and 2019-21, India.**

**Fig A8. Women**'**s exposure to SIPV by their age, education, and age at first cohabitation in 2005-06, 2015-16, and 2019-21, India.**

(a) (b)

(c)

**Fig A9. Women**'**s exposure to SIPV by their employment status, access to bank account, and media exposure in 2005-06, 2015-16, and 2019-21, India.**

(a) (b)

(c)

**Fig A10. Women**'**s exposure to SIPV by their experience of interparental violence and husband’s education in 2005-06, 2015-16, and 2019-21, India.**

(a) (b)

**Fig A11. Women**'**s exposure to SIPV by religion, social group, household wealth, and place of residence in 2005-06, 2015-16, and 2019-21, India.**

(a) (b)

(c) (d)

**Fig A12. Women**'**s exposure to SIPV by different states and union territories, in 2005-06, 2015-16, and 2019-21, India.**


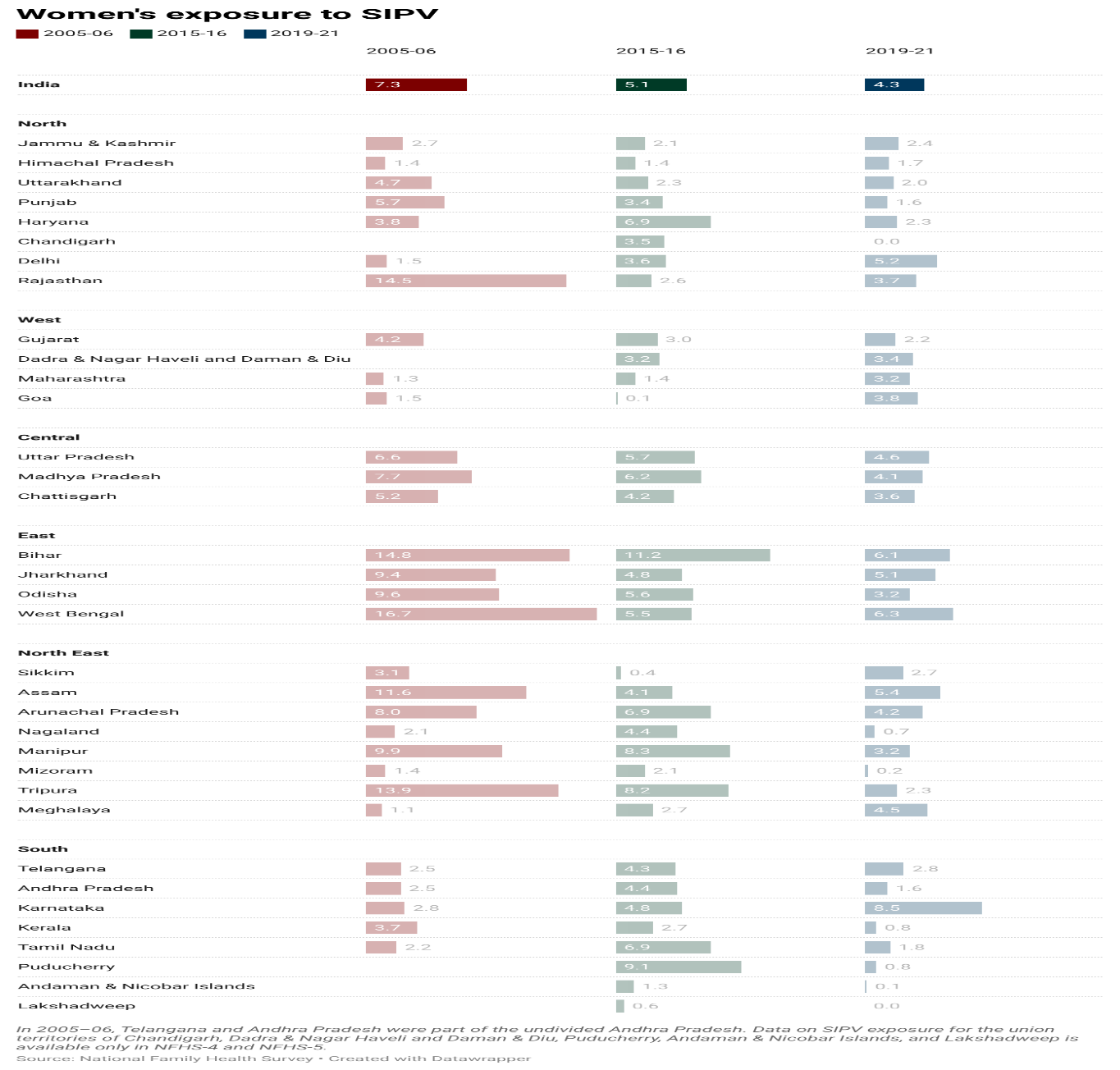


**Fig A13. Women**'**s exposure to EIPV in 2005-06, 2015-16, and 2019-21, India.**

**Fig A14. Women**'**s exposure to EIPV by their age, education, and age at first cohabitation in 2005-06, 2015-16, and 2019-21, India.**

(a) (b)

(c)

**Fig A15. Women**'**s exposure to EIPV by their employment status, access to bank account, and media exposure in 2005-06, 2015-16, and 2019-21, India.**

(a) (b)

(c)

**Fig A16. Women**'**s exposure to EIPV by their experience of interparental violence and husband’s education in 2005-06, 2015-16, and 2019-21, India.**

(a) (b)

**Fig A17. Women**'**s exposure to EIPV by religion, social group, household wealth, and place of residence in 2005-06, 2015-16, and 2019-21, India.**

(a) (b)

(c) (d)

**Fig A18. Women**'**s exposure to EIPV by different states and union territories, in 2005-06, 2015-16, and 2019-21, India.**


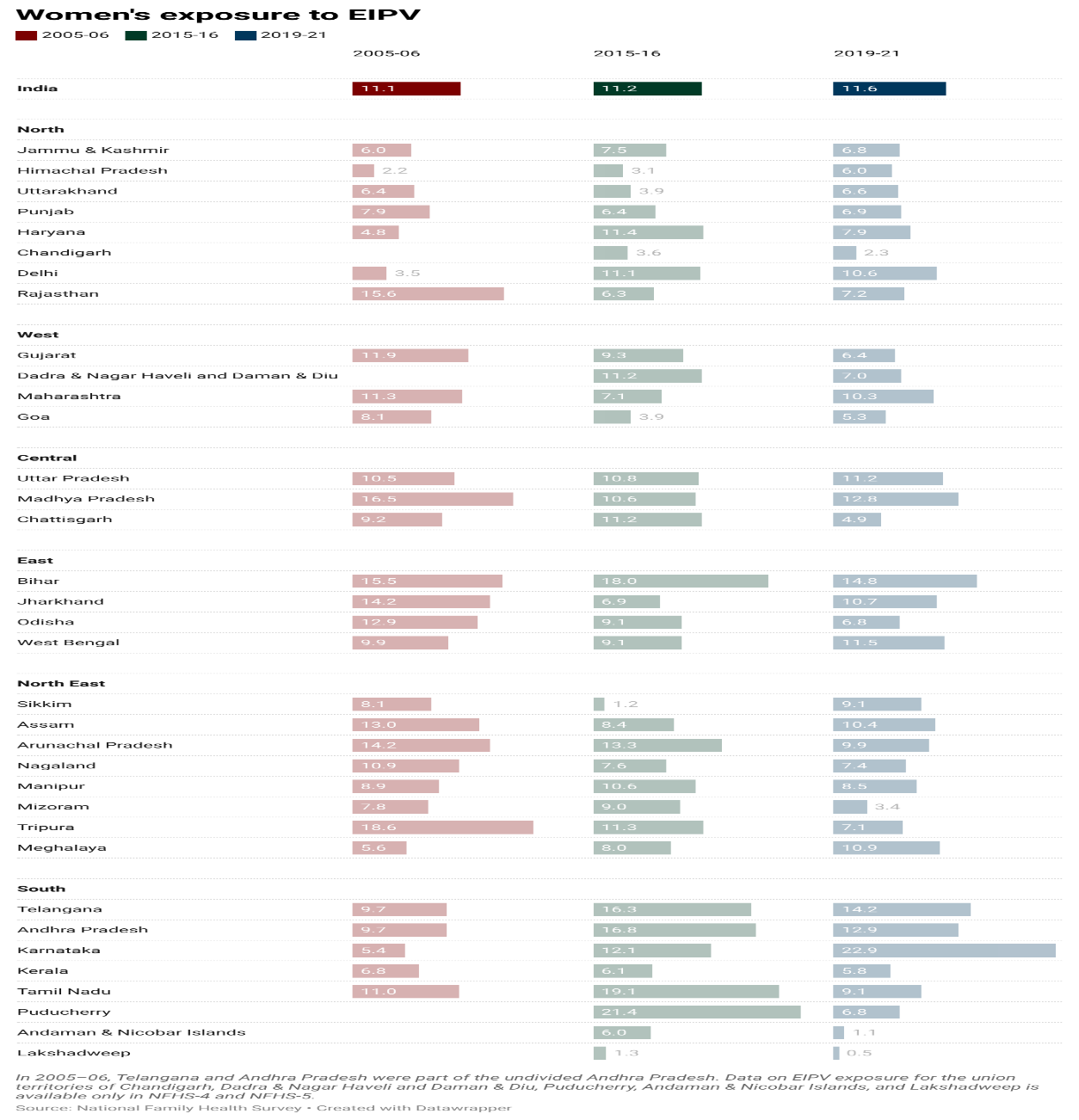

Supplement: S1 Appendix — (DOCX) [file pone.0318350.s001.docx]
